# CA125/MUC16 Is Dispensable for Mouse Development and Reproduction

**DOI:** 10.1371/journal.pone.0004675

**Published:** 2009-03-05

**Authors:** Dong-Joo Cheon, Ying Wang, Jian Min Deng, Zhen Lu, Lianchun Xiao, Chun-Ming Chen, Robert C. Bast, Richard R. Behringer

**Affiliations:** 1 Program in Genes and Development, The University of Texas Graduate School of Biomedical Sciences at Houston, Houston, Texas, United States of America; 2 Department of Genetics, University of Texas M. D. Anderson Cancer Center, Houston, Texas, United States of America; 3 Department of Experimental Therapeutics, University of Texas M. D. Anderson Cancer Center, Houston, Texas, United States of America; 4 Department of Biostatistics, University of Texas M. D. Anderson Cancer Center, Houston, Texas, United States of America; Dresden University of Technology, Germany

## Abstract

Cancer antigen 125 (CA125) is a blood biomarker that is routinely used to monitor the progression of human epithelial ovarian cancer (EOC) and is encoded by *MUC16*, a member of the mucin gene family. The biological function of CA125/MUC16 and its potential role in EOC are poorly understood. Here we report the targeted disruption of the of the *Muc16* gene in the mouse. To generate *Muc16* knockout mice, 6.0 kb was deleted that included the majority of exon 3 and a portion of intron 3 and replaced with a *lacZ* reporter cassette. Loss of *Muc16* protein expression suggests that *Muc16* homozygous mutant mice are null mutants. *Muc16* homozygous mutant mice are viable, fertile, and develop normally. Histological analysis shows that *Muc16* homozygous mutant tissues are normal. By the age of 1 year, *Muc16* homozygous mutant mice appear normal. Downregulation of transcripts from another mucin gene (*Muc1*) was detected in the *Muc16* homozygous mutant uterus. Lack of any prominent abnormal phenotype in these *Muc16* knockout mice suggests that CA125/MUC16 is not required for normal development or reproduction. These knockout mice provide a unique platform for future studies to identify the role of CA125/MUC16 in organ homeostasis and ovarian cancer.

## Introduction

CA125 is a tumor antigen that is routinely used to detect and monitor the growth of ovarian carcinoma and the outcome of treatment of ovarian cancer patients [1]. CA125 is a high molecular weight (>1 M Dalton) mucin-type glycoprotein encoded by the *MUC16* gene [2], [3], [4], [5]. MUC16 consists of 22,097 amino acid residues; the gene spans 174 kb on human chromosome 19. MUC16 contains a large extracellular domain followed by a tandem repeat domain, a transmembrane domain and a short cytosolic tail [2], [3]. MUC16 is expressed at the apical surface of coelomic epithelia and its derivatives including epithelial cells in the Müllerian duct, fallopian tube, endometrium, endocervix, and mesothelial cells lining the peritoneal and pleural cavities [6], [7], [8].

In spite of the utility of CA125 in the clinic, the biological functions of CA125/MUC16 in normal physiology and ovarian cancer are still poorly understood. CA125 serum levels are also elevated in some benign conditions and other carcinomas [1], raising the possibility that MUC16 might have broad biological functions. Based on the structural similarity of MUC16 to other mucins, it is likely that MUC16 may have functions that are similar to other mucins [reviewed in 9]. MUC16 has been shown to play roles in mediating both anti-adhesion and cell adhesion, and in suppressing the immune system. The anti-adhesive property of MUC16 [10] has been suggested to provide a protective barrier for the epithelial surface from bacterial adherence [11] and mechanical injury [12]. Furthermore, MUC16 in the endometrium of the uterus has been shown to prevent the attachment of trophoblast during non-receptive status [13]. In contrast, MUC16 has also been demonstrated to mediate cell adhesion by binding to other cell surface glycoproteins such as galectin-1 and mesothelin [14], [15]. The binding of MUC16 to mesothelin is of particular interest because this interaction implicates a role for MUC16 in dissemination of ovarian cancer cells to the peritoneal cavity [15]. MUC16 has been shown to suppress the immune system, by suppressing the activity of natural killer cells [16], [17]. Immune suppressive properties of MUC16, along with its expression in ovarian cancer cells and fetal periderm, suggest a role for MUC16 in immune evasion of ovarian cancer cells from the host immune system and in fetal tolerance from maternal immune rejection during early pregnancy [12], [16], [18]. Other studies suggest roles for MUC16 in endometriosis, secretion, and other tissue-specific functions such as in the upper respiratory tract [12], [19], [20], [21]. However, biological functions of MUC16 in normal physiology as well as in pathological conditions such as ovarian cancer still remain unknown. This is largely due to the lack of *in vivo* models that disrupt MUC16 expression.

Previously, we characterized the mouse *Muc16* homolog by means of gene structure and expression pattern, demonstrating that mouse *Muc16* is the ortholog of the human *MUC16* gene [22]. Here we generated *Muc16* mutant mice using gene targeting in mouse embryonic stem (ES) cells. *Muc16* homozygous mutant mice are normal and fertile, and do not display abnormal phenotypes by 1 year of age. Lack of abnormal phenotypes in these *Muc16* knockout mice suggests that CA125/MUC16 is not required for normal development and reproduction. Our mice provide a valuable *in vivo* model system to explore MUC16 function in other physiological and pathological conditions such as ovarian carcinogenesis.

## Methods

### Disruption of the mouse *Muc16* gene in ES cells

The gene structure of the mouse *Muc16* gene was determined as described in a previous study [22]. The targeting vector was generated from 129S7/AB2.2 mouse bacterial artificial chromosome (BAC) clones (Sanger Institute, UK; Ensemble ID #s: bMQ-301I10 and bMQ-216K15) containing genomic fragments of the mouse *Muc16* locus.

A 6.1-kb region of *Muc16* homology was used. A 4-kb *Ssp*F/*Bgl*1 fragment spanning *Muc16* intron 2 and extending 580 bp into exon 3 was used as a 5′ arm of homology, whereas a 2.1-kb *Bcl*I/*Sca*I fragment spanning *Muc16* intron 3 was used as a 3′ arm of homology. The 5′ and 3′ arms of homology were inserted into a pBluescript II KS(−) vector (Stratagene, La Jolla, CA) using *Kpn*I/*Sal*I sites and *Not*I/*Sac*II sites, respectively.

**Figure 1:**
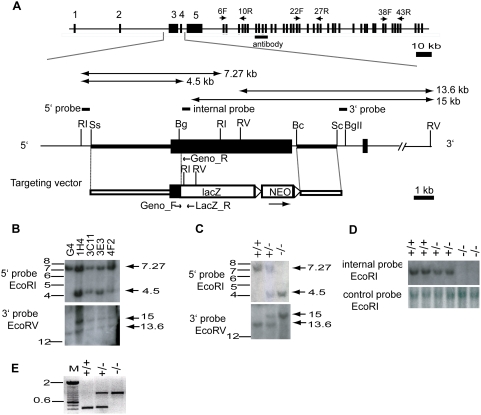
Targeted disruption of the mouse *Muc16* gene. (A) Strategy used for *Muc16* gene targeting. Organization of the mouse *Muc16* gene, structure of the targeted allele, targeting vector, location of Southern probes, protein region recognized by anti-mouse MUC16 polyclonal antibody, primers for RT-PCR (6F, 10R; 22F, 27R; 38F, 43R), and genotyping are shown. Bc, *Bcl*I; Bg, *Bgl*I; BgII, *Bgl*II; RI, *Eco*RI; RV, *Eco*RV; Sc, *Sca*I; Ss, *Ssp*F, lacZ, IRES-lacZ-pA; NEO, PgkneobpA; triangle, *loxP* sites. (B) Southern analysis of genomic DNA isolated from ES cell clones. The sizes of the restriction fragments detected by 5′ and 3′ probes are indicated. (C) Southern analysis of genomic DNA isolated from representative pups. +/+, wild type; +/−, heterozygote; −/−, homozygous mutant. (D) Targeted deletion of exon 3 region of the mouse *Muc16* locus. Loss of exon 3 region is detected by an internal probe in the homozygous mutants. (E) PCR analysis to determine genotypes. A 414-bp product and a 800-bp product are amplified by the wild-type primers and the mutant primers, respectively.

An ‘IRES-T-lacZ’ reporter cassette, followed by a PgkneobpA expression cassette flanked by loxP sites [23] was inserted between the two arms of homology using *Sal*I/*Not*I sites, replacing most of *Muc16* exon 3 leaving the initial 580-bp of exon 3 intact. The final targeting vector (Figure 1) was linearized with *Sac*II at a unique site in the vector backbone. 20–25 µg of the linearized targeting vector was electroporated into 10^7^ cells of the G4 129S6B6F1 hybrid ES cell line [24] as described [25]. A total of 372 G418-resistant ES cell clones were initially screened by *Eco*RI digestion and hybridized with a 5′ probe external to the region of homology. Four of the 372 (1.1%) G418-resistant ES cell clones were identified as correctly targeted and confirmed using a 3′ external probe.

### Generation of chimeric mice and germline transmission of *Muc16* mutant allele

*Muc16* mutant ES cell clones were microinjected into albino C57BL/6 (B6) blastocysts and the resulting embryos were transferred to the uterine horn of 2.5 days post coitus (dpc) pseudopregnant foster mothers, according to standard protocol [25]. Chimeric males were crossed with B6 females, and the agouti-colored offspring were analyzed for germline transmission of the *Muc16* mutant allele. Tail DNA from the agouti pups was analyzed by Southern blot analysis using 5′ and 3′ probes to identify *Muc16* heterozygous mutants. We also devised a PCR genotyping strategy. Primers used for PCR genotyping (Geno_F, Geno_R, and LacZ_R) are shown in Table 1 and Figure 1A. PCR conditions were 35 cycles at 95°C for 40 sec, 57°C for 1 min., and 72°C for 1 min. This results in a 414-bp wild-type band and an 800-bp mutant band (Figure 1E). *Muc16* heterozygous mutants were intercrossed to generate *Muc16* homozygous mutants. Wild-type siblings were used as controls. All *Muc16* mutant animals were analyzed on a B6/129 mixed genetic background. All experimental animals were maintained in accordance with the Institutional Animal Care and Use Committee of the M.D. Anderson Cancer Center and the NIH Guide for the Care and Use of Laboratory animals.

**Table 1:** Summary of PCR primer sequences, annealing temperatures, and expected product sizes.

Primers	Sequence of forward(F) and reverse(R) primers	Annealing temperature (°C)	Product size (bp)	Accession #
6F10R_F	5′-GCCCATGTTCAAGAATAGCAGTATTGG-3′	56	698	XM_911929
6F10R_R	5′-GTAGCAGAGAGGGGCTTGTGGTTG-3′	58		
22F27R_F	5′-CACAACATTACTCAACTGGGTCCC-3′	53	855	XM_911929
22F27R_R	5′-GTTCACTGTGAACAGCTCCTC-3′	52		
38F43R_F	5′-CCCTTGGTCCAGAATGAATCCC-3′	53	574	XM_911929
38F43R_R	5′-CACGTGCAGATCTTTCAACTGGTAGG-3′	58		
Geno_F	5′-CAGCATTTCCTATCCAATCACTAACCAG-3′	56	414	XM_911929
Geno_R	5′-CTAGATTGTATCTGAGCTGTGGTCC-3′	55		
Geno_F	5′-CAGCATTTCCTATCCAATCACTAACCAG-3′	56	800	XM_911929
LacZ_R	5′-GAAACCAGGCAAAGCGCCATTC-3′	56		
Muc1_F	5′-CCTACCATCCTATGAGTGAATACC-3′	51	119	NM_013605
Muc1_R	5′-GAGACTGCTACTGCCATTACCTG-3′	55		
Muc4_F	5′-CCTTCACTGATAACCGCTGCTT-3′	55	115	NM_080457
Muc4_R	5′-GCGGAGGCATTTTCATCCT-3′	52		
Meso_F	5′-GGTCCTGTGGAAGTCCCATCTG-3′	56	129	NM_018857
Meso_R	5′-CTTGTCTTTGTAGTCTGGGTCTGC-3′	57		

### RT-PCR analysis of *Muc16* transcripts

Total RNA was extracted from adult tissues of wild-type and homozygous mutant animals using Trizol® reagent (Life Technologies, Carlsbad, CA), according to the manufacturer's instructions. All procedures of cDNA synthesis and RT-PCR analysis were performed as described [22]. Briefly, cDNA was synthesized by reverse transcription (RT) from 1 µg of total RNA using random hexamers and the SuperScript II first-strand synthesis system for RT-PCR (Invitrogen, Carlsbad, CA). One microliter of the first-strand cDNA was used as the template for the PCR reactions. Primer sequences used in this study are shown in Table 1 and Supporting Table S1. For *Muc16* RT-PCR (6F, 10R; 22F, 27R and 38F, 43R), PCR conditions were 30 cycles at 94°C for 30 sec, 55°C for 45 sec, and 72°C for 1 min. β-actin RT-PCR was performed as an internal control. RT-PCR of adult ovary cDNA in the absence of reverse transcriptase served as a negative control.

### Histological analysis and MUC16 immunofluorescence

Generation of rabbit anti-mouse MUC16 polyclonal antibody was described previously [22]. The region of the MUC16 protein used to generate the polyclonal antibody is shown in Figure 1. Tissues were fixed in 4% paraformaldyhyde (PFA) in phosphate-buffered saline (PBS) at 4°C overnight, then dehydrated, embedded in paraffin, and sectioned at 5 µm. MUC16 immunofluorescence was performed as described previously [22]. Briefly, antigen retrieval was performed in 50 mM Tris buffer (pH 9.0) for 20 minutes, then blocking was performed using 10% normal goat serum diluted with PBS and 0.05% Tween-20 (PBS-T) for 1 hour at room temperature. The slides were incubated with anti-mouse MUC16 antibody (1∶50) overnight at 4°C and then incubated with the secondary biotinylated antibody (1∶250) (Vector Laboratories, Inc., Burlingame, CA) for 30 minutes at room temperature. The slides were next incubated with fluorescein avidin D (1∶300) (Vector Laboratories, Inc., Burlingame, CA) for another 30 minutes, washed, counterstained with VECTASHIELD mounting media with DAPI (Vector Laboratories, Inc., Burlingame, CA), and examined using fluorescence microscopy.

### Real-time reverse transcription-PCR (qRT-PCR)

Total RNA was extracted from adult tissues of *Muc16* wild-type and homozygous mutants using Trizol® reagent (Life Technologies, Carlsbad, CA). All RNA was treated with DNase I (Amplification Grade, Invitrogen, Carlsbad, CA). RNA (0.5 µg) was reverse transcribed using the SuperScript II first-strand synthesis system for RT-PCR (Invitrogen, Carlsbad, CA) with random primers. Three microliters of the synthesized cDNA were mixed with SYBR® Green PCR master mix (Applied Biosystems, Foster city, CA) and qRT-PCR was performed on an ABI PRISM 7900HT (Applied Biosystems, Foster city, CA). The expression level of each amplicon was calculated by normalizing each cDNA to glyceraldehyde 3-phosphate dehydrogenase (*Gadph*) and then calculating fold change compared with the control. All experiments were repeated three times.

### Statistical analysis

Statistical analysis was performed using SAS (SAS Institute Inc.) or Splus (Insightful Corp.) statistical packages. For the viability analysis, a chi-square analysis was performed to calculate statistical significance. For the fertility analysis, the Wilcoxon rank sum test was used to compare the number of pups per litter between different genotypes. For the growth data, a mixed model with repeat approach was fitted to estimate genotype effect on mouse growth. The Wilcoxon rank sum test was used to compare the expression levels of *Muc1*, *Muc4* and *Mesothelin* (*Msln*) between the wild-type and the homozygous mutant mice.

## Results

### Targeted disruption of mouse *Muc16* gene

To understand the biological function of CA125/MUC16, we disrupted the mouse *Muc16* gene (Figure 1A). To simultaneously knockout and mark the *Muc16* gene, a 6-kb fragment was deleted coding for the majority of exon 3 and a portion of intron 3 and replaced with a IRES-T-lacZpA-loxP-PGKneobpA-loxP reporter cassette (Figure 1A). This exon was chosen because it showed sequence homology to exon 1 of human *MUC16* that we confirmed by DNA sequencing. The gene targeting strategy employed a total of 6.1-kb homology and the arms of homology were amplified by PCR from 129S7/AB2.2 mouse genomic BAC clones. Of 372 G418-resistant ES cell clones, 4 clones were identified as being correctly targeted (Figure 1B). Two different ES cell clones (1H4, 3C11) were injected into blastocysts independently and gave rise to germline-transmitting chimeric males. Heterozygous offspring were intercrossed to generate homozygous mutant progeny (Figure 1C). Genotypes of offspring were confirmed by Southern blotting (Figure 1C). To further demonstrate that coding exon 3 was disrupted, we designed an internal probe against the deleted sequence (Figure 1A) and performed Southern blot analysis. As expected, the predicted exon 3 sequence was deleted in homozygous mutants (Figure 1D). To accelerate the genotyping process, we also devised an allele-specific PCR genotyping strategy, amplifying a 414-bp wild-type band and a 800-bp mutant band (Figure 1E). Both female and male mutant mice showed the same PCR band patterns. Unfortunately, both heterozygotes and homozygotes carrying the *lacZ* allele did not express β-galactosidase protein or activity by immunofluorescence or X-gal staining, respectively (data not shown). RT-PCR analysis showed that *Muc16*-*lacZ* chimeric transcripts and downstream *lacZ* transcripts were barely or not detected in the mutant mice (Supporting Text S1, Supporting Figure S1B).

### *Muc16*/MUC16 *expression in Muc16* homozygous mutants

**Figure 2:**
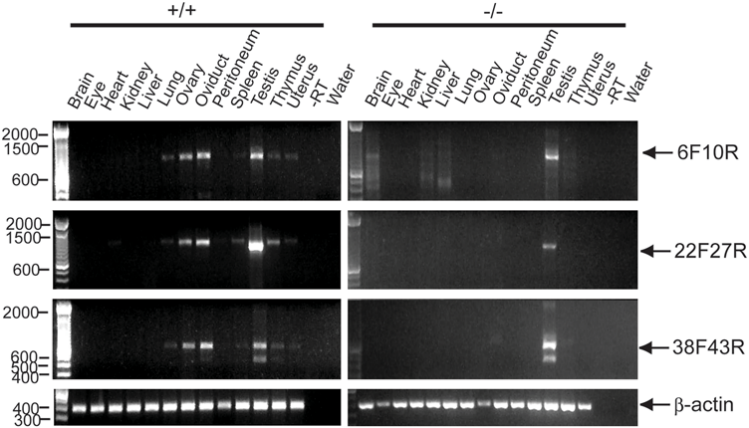
RT-PCR analysis of adult *Muc16* wild-type (+/+) and homozygous mutant (−/−) tissues. Primers used for RT-PCR analysis are shown in Table 1 and Figure 1A. *Muc16* amplified products are indicated by arrows. The reactions without reverse transcriptase (−RT) and without cDNA template (water) serve as negative controls.

To determine the effect on *Muc16*/MUC16 expression in homozygous mutants, we analyzed the expression of *Muc16* mRNA and protein by RT-PCR and immunofluorescence, respectively. For RT-PCR, three primer sets were used that can detect *Muc16* transcripts from different subregions of the *Muc16* gene (Figure 1A). *Muc16* transcripts were not detected in *Muc16* homozygous mutant tissues except for the testis that was positive for all three subregions (Figure 2). Sequence analysis verified that these homozygous mutant testis-derived RT-PCR fragments were identical to wild type. Primers used to amplify the region between exons 38 and 43 yielded a long and short isoform in both wild-type and homozygous mutant testes (Figure 2). Sequence analysis of these two isoforms demonstrated that the size differences were limited to exon 39, suggesting complex RNA processing in this region (data not shown). A comprehensive RT-PCR analysis of *Muc16* transcripts generated from the testes of *Muc16* homozygous mutants suggests that the expression of exon 3 is eliminated or significantly reduced, depending on the region of exon 3 examined (Supporting Text S1, Supporting Figure S1). Thus, no transcripts containing the entire exon 3 coding region can be generated from the targeted locus in homozygous mutant testes.

**Figure 3:**
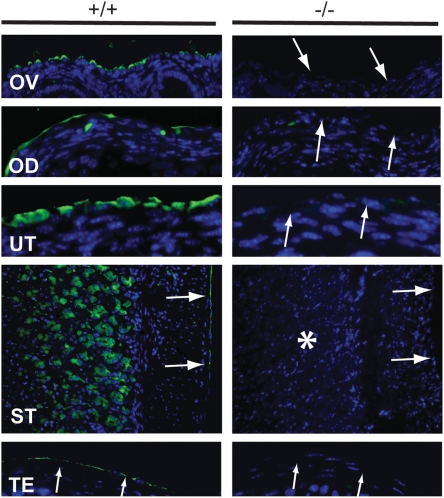
Immunohistochemistry of adult *Muc16* wild-type (+/+) and homozygous mutant (−/−) tissues. MUC16 protein (arrows) was absent in *Muc16* −/− tissues. Asterisk indicates the loss of MUC16 protein in the chief cells in the stomach. OD, oviduct; OV, ovary; ST, stomach; TE, testis; UT, uterus. Scale bar = 50 µm.

We also examined MUC16 expression by immunofluorescence. In wild-type tissues, MUC16 protein was detected in the mesothelia of various organs (Figure 3). In contrast, MUC16 protein was absent in mesothelia as well as other cell types such as chief cells in the stomach (Figure 3). Although we detected *Muc16* transcripts in homozygous mutant testes by RT-PCR there was no detectable MUC16 protein detected by immunofluorescence in the mesothelia surrounding the testes (Figure 3). We conclude that these testicular transcripts are not translated, at least in the region detected by the polyclonal antibody, perhaps because of an upstream frameshift in the region of exon 3. Loss of intact *Muc16* transcripts and protein in *Muc16* homozygous mutant tissues suggests that the *Muc16* homozygous mutants are null mutants. We observed this same absence of expression in tissues from homozygous mutant parents and their offspring.

### *Muc16* homozygous mutant mice are viable, fertile and grow normally

**Figure 4:**
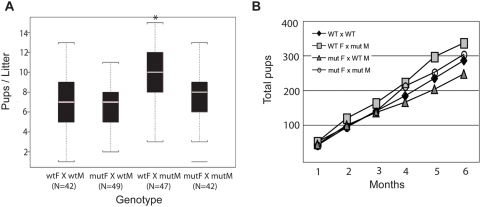
Fertility analysis of *Muc16* wild-type (wt) and homozygous mutant (mut) mice. Seven breeding pairs of the indicated genotypes (M, male; F, female) were mated for 6 months and the numbers of pups were recorded. (A) The median pups per litter is shown with the upper and lower edges of the boxes equal to the 25% and 75% quartiles. The whiskers indicate 5% and 95% quartiles. Total numbers (N) of litters are shown under each type of breeding pair. Asterisk indicates a statistical significance in wt F×mut M pairs, compared to wt pairs (p<0.0001). But no statistical differences were found in mut F×wt M and mut F×mut M pairs, compared to wt pairs (p = 0.33, 0.61). (B) The total accumulated pups for each type of breeding pair was counted for 6 months.

To determine the phenotype of *Muc16* homozygous mutants, we intercrossed *Muc16* heterozygotes. We obtained wild-type, heterozygous, and homozygous mutants according to the Mendelian ratio (69 +/+; 137 +/−; 64 −/−). *Muc16* homozygous mutants were grossly normal and showed a normal rate of growth (data not shown). Because MUC16 is implicated in reproduction, we monitored the fertility of *Muc16* homozygous mutants for 6 months. Both *Muc16* homozygous mutant males and females were fertile (Figure 4). Interestingly, *Muc16* homozygous mutant males bred with wild-type females yielded significantly more progeny in this breeding assay compared to wild-type males bred with wild-type females (Figure 4). *Muc16* expression in testes is only found in mesothelia surrounding the gonad [22]. Thus, the reason for this difference is not clear.

**Figure 5:**
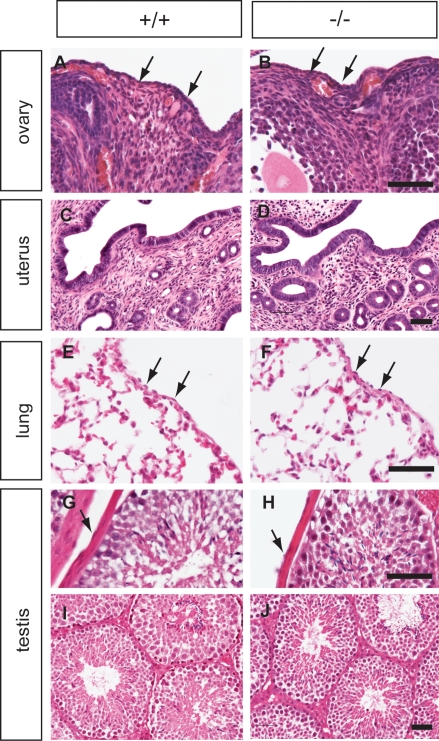
Histology of *Muc16* wild type (+/+) and homozygous mutant (−/−) tissues. The ovary (A, B), uterus (C, D), lung (E, F), and testis (G–J) from wild-type (left panel) and homozygous mutant animals (right panel) were analyzed by H&E staining. Tissues were collected at 3 months (A–B, E–J) and 1 year of age (C, D). Arrowheads indicate mesothelial cells. Scale bars = 50 µm.

We did not detect any histological differences between wild-type and homozygous mutant tissues (Figure 5). Up to 1 year, *Muc16* homozygous mutant mice were normal both in appearance and histology (Figure 5). Similar results were obtained for a second *Muc16 lacZ* mutant mouse line generated from an independently targeted ES cell clone (data not shown).

### Expression of *Muc1*, *Muc4*, and *Msln* in *Muc16* homozygous mutants

**Figure 6:**
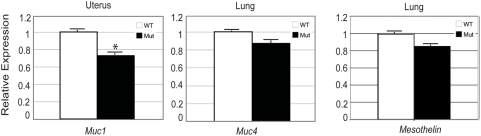
Real-time reverse transcription-PCR (qRT-PCR) of *Muc1*, *Muc4*, and *Msln* in *Muc16* wild-type (WT) and homozygous mutant (Mut) tissues. Values are expressed mean +/− s.e.m. and the mean of the wild type set to “1”. Asterisk indicates significant downregulation of *Muc1* (p = 0.01) in *Muc16* homozygous mutant uterus.

One possible explanation why *Muc16* homozygous mutants were normal would be functional compensation by other mucin genes. To test this idea, we determined the expression level of *Muc1* and *Muc4* in *Muc16* homozygous uterus and the lung, respectively. We chose *Muc1* and *Muc4* because the expression pattern of these mucin genes overlapped with *Muc16* and *Muc16* mRNA levels are increased in the lung of *Muc4* knockout mice [26, Wanda O'Neal, personal communication]. The expression levels of *Muc1* but not *Muc4* were significantly downregulated in the *Muc16* homozygous mutant tissues (Figure 6, *Muc1* p = 0.01). These results suggest that MUC16 is required to maintain normal transcript levels of *Muc1*. *Msln* transcript levels were not significantly different in lung between *Muc16* homozygous mutants and wild-type controls (Figure 6).

## Discussion

CA125/MUC16 has served as a very useful serum marker to monitor the progression of ovarian cancer or response to treatment. Expression of CA125/MUC16 in multiple normal tissues and in different pathological and physiological conditions implies a broad biological role for CA125/MUC16 [1]. This study reports that *Muc16* knockout mice appear normal and do not display any prominent abnormal phenotypes by 1 year of age. The phenotype of these *Muc16* knockout mice is consistent with that of other mucin mutant mice. Except for *Muc2* knockout mice, *Muc1* and *Muc4* knockout mice were normal and fertile [27], [28, Wanda O'Neal, personal communication]. Based on the fact that there are more than 17 mucin genes, whose expression patterns largely overlap in different tissues [9], it is possible that functional redundancy can compensate for the loss of other mucin genes. Although we did not observe upregulation of *Muc1* and *Muc4* genes in *Muc16* homozygous mutant tissues, it is still possible that other mucin genes might compensate for *Muc16* loss. Another possible reason why *Muc16* homozygous mutant mice are normal might be the functional compensation by mesothelin in the mesothelia. Mesothelin is a GPI-linked cell surface glycoprotein, abundantly expressed in normal mesothelial cells [29], where MUC16 is also expressed [22]. However, we did not detect any significant difference in *Msln* expression levels in *Muc16* homozygous mutant lung.

In conclusion, we provide *in vivo* evidence suggesting that CA125/MUC16 is not required for normal mouse development and reproduction. Because *Muc16* knockout mice are fertile, they can be bred with other mouse lines to identify functional interactions with other genes. In addition, mesothelial cell lines generated from *Muc16* homozygous mutant mice may provide useful reagents for *in vitro* studies. These mice will also be a useful resource for future studies to provide insights into the role of MUC16 in organ homeostasis and ovarian cancer both *in vitro* and *in vivo*.

## Supporting Information

Text S1 RT-PCR analysis of Muc16 homozygous mutant testes. All of the adult tissues screened by RT-PCR in the Muc16 homozygous mutants were negative for 3 different regions of the Muc16 locus except for the testes (Figure 2). To understand the transcripts generated by the Muc16-targeted allele from homozygous mutant testes, we performed RT-PCR, using various sets of primers (Supporting Table S1, Supporting Figure S1A). A robust band of the correct size was detected using primers for exons 1 and 3 in wild-type and homozygous mutant testes (Supporting Figure S1B). This suggests that transcription from the Muc16 targeted locus to generate mRNA containing exons 1 to 3 appears to be normal. Using an exon 3 primer present in both the wild-type and null alleles and an exon 3 primer located within the exon 3 deleted region of the targeted allele, we detected a positive signal in wild-type but not homozygous mutant testes (Supporting Figure S1B). These results support our Southern analysis that indeed the majority of exon 3 has been deleted by our targeting strategy (Figure 1D). Thus, the targeted allele cannot generate exon 3-containing transcripts for the region that was deleted. To determine if Muc16-lacZ chimeric transcripts were generated, we used exon 3 and lacZ primers. Muc16-lacZ chimeric transcripts were detected from the targeted Muc16 allele but the signal was very weak (Supporting Figure S1B). In addition, using lacZ primers, lacZ transcripts downstream of the Muc16-lacZ fusion were undetectable (Supporting Figure S1B). This suggests that Muc16-lacZ chimeric transcripts may be very unstable, leading to insufficient production of β-galactosidase for detection by immunofluorescence and X-gal staining (data not shown). We also performed RT-PCR using primers for exons 2 and 4 in case exon 3 which is very large was skipped by alternative splicing, however, no signal of the predicted size was detected (data not shown). Even if exon 3 was skipped it would lead to a frameshift and no MUC16 protein should be generated. We also performed RT-PCR using primers located in exons 4 and 5, and exons 5 and 6. Both sets of primers amplified the predicted sized bands in both wild-type and homozygous mutant testes (Supporting Figure S1C). This suggests that exons 4–6 are being transcribed in the mutant. The neo gene has its own promoter (Pgk) for expression in mouse ES cells. It is possible that there may be readthrough of the pA signal from the neo cassette. Therefore, we also used primers for neo and exon 4. No signal was detected in homozygous mutant testes (Supporting Figure S1B). This suggests that the neo pA signal is functional. Finally, we repeated the RT-PCR using primers for exons 6 and 10 located downstream of the exon 3 targeted modification. A robust signal was detected in wild-type testes and a detectable though weaker signal in homozygous mutant testes (Supporting Figure S1B). These results are similar to our initial survey of expression (Figure 2). Taken together, these results suggest that the targeted allele does not express the full complement of transcripts generated by the wild-type allele. In addition, no MUC16 protein was detected by immunofluorescence using a polyclonal antibody (Figure 3). With respect to the testis, the targeted modification is clearly a loss-of-function allele. Formal demonstration that the targeted mutation is a null allele in the testis is hampered because Muc16 is a very large gene with many exons [22].
(0.03 MB DOC)

Table S1 Summary of PCR primer sequences.
(0.04 MB DOC)

Figure S1 RT-PCR analysis of Muc16 locus. (A) Organization of the mouse Muc16 gene and location of primers used for RT-PCR analysis (indicated by arrows). (B, C) RT-PCR analysis of Muc16 expression upstream and downstream of the targeted region in adult Muc16 wild-type and null testis. Forward (F) and reverse (R) primers are indicated.
(2.08 MB TIF)
