# Supplementary material for: CA125/MUC16 Is Dispensable for Mouse Development and Reproduction
Source: PLoS One. 2009 Mar 5;4(3):e4675. doi: 10.1371/journal.pone.0004675 (PMC2650410; doi:10.1371/journal.pone.0004675)
Supplement: Text S1 — RT-PCR analysis of Muc16 homozygous mutant testes. All of the adult tissues screened by RT-PCR in the Muc16 homozygous mutants were negative for 3 different regions of the Muc16 locus except for the testes (Figure 2). To understand the transcripts generated by the Muc16-targeted allele from homozygous mutant testes, we performed RT-PCR, using various sets of primers (Supporting Table S1, Supporting Figure S1A). A robust band of the correct size was detected using primers for exons 1 and 3 in wild-type and homozygous mutant testes (Supporting Figure S1B). This suggests that transcription from the Muc16 targeted locus to generate mRNA containing exons 1 to 3 appears to be normal. Using an exon 3 primer present in both the wild-type and null alleles and an exon 3 primer located within the exon 3 deleted region of the targeted allele, we detected a positive signal in wild-type but not homozygous mutant testes (Supporting Figure S1B). These results support our Southern analysis that indeed the majority of exon 3 has been deleted by our targeting strategy (Figure 1D). Thus, the targeted allele cannot generate exon 3-containing transcripts for the region that was deleted. To determine if Muc16-lacZ chimeric transcripts were generated, we used exon 3 and lacZ primers. Muc16-lacZ chimeric transcripts were detected from the targeted Muc16 allele but the signal was very weak (Supporting Figure S1B). In addition, using lacZ primers, lacZ transcripts downstream of the Muc16-lacZ fusion were undetectable (Supporting Figure S1B). This suggests that Muc16-lacZ chimeric transcripts may be very unstable, leading to insufficient production of β-galactosidase for detection by immunofluorescence and X-gal staining (data not shown). We also performed RT-PCR using primers for exons 2 and 4 in case exon 3 which is very large was skipped by alternative splicing, however, no signal of the predicted size was detected (data not shown). Even if exon 3 was skipped it would lead to a frameshift a [file pone.0004675.s001.doc]

**Supporting Text S1. RT-PCR analysis of *Muc16* homozygous mutant testes**

All of the adult tissues screened by RT-PCR in the *Muc16* homozygous mutants were negative for 3 different regions of the *Muc16* locus except for the testes (**Figure 2**). To understand the transcripts generated by the *Muc16*-targeted allele from homozygous mutant testes, we performed RT-PCR, using various sets of primers (**Supporting Table S1, Supporting Figure S1A**). A robust band of the correct size was detected using primers for exons 1 and 3 in wild-type and homozygous mutant testes (**Supporting Figure S1B**). This suggests that transcription from the *Muc16* targeted locus to generate mRNA containing exons 1 to 3 appears to be normal. Using an exon 3 primer present in both the wild-type and null alleles and an exon 3 primer located within the exon 3 deleted region of the targeted allele, we detected a positive signal in wild-type but not homozygous mutant testes (**Supporting Figure S1B**). These results support our Southern analysis that indeed the majority of exon 3 has been deleted by our targeting strategy (**Figure 1D**). Thus, the targeted allele cannot generate exon 3-containing transcripts for the region that was deleted. To determine if *Muc16-lacZ* chimeric transcripts were generated, we used exon 3 and *lacZ* primers. *Muc16-lacZ* chimeric transcripts were detected from the targeted *Muc16* allele but the signal was very weak (**Supporting Figure S1B**). In addition, using *lacZ* primers, *lacZ* transcripts downstream of the *Muc16-lacZ* fusion were undetectable (**Supporting Figure S1B**). This suggests that *Muc16-lacZ* chimeric transcripts may be very unstable, leading to insufficient production of β-galactosidase for detection by immunofluorescence and X-gal staining (**data not shown**). We also performed RT-PCR using primers for exons 2 and 4 in case exon 3 which is very large was skipped by alternative splicing, however, no signal of the predicted size was detected (**data not shown**). Even if exon 3 was skipped it would lead to a frameshift and no MUC16 protein should be generated. We also performed RT-PCR using primers located in exons 4 and 5, and exons 5 and 6. Both sets of primers amplified the predicted sized bands in both wild-type and homozygous mutant testes (**Supporting Figure S1C**). This suggests that exons 4-6 are being transcribed in the mutant. The *neo* gene has its own promoter (*Pgk*) for expression in mouse ES cells. It is possible that there may be readthrough of the pA signal from the *neo* cassette. Therefore, we also used primers for *neo* and exon 4. No signal was detected in homozygous mutant testes (**Supporting Figure S1B**). This suggests that the *neo* pA signal is functional. Finally, we repeated the RT-PCR using primers for exons 6 and 10 located downstream of the exon 3 targeted modification. A robust signal was detected in wild-type testes and a detectable though weaker signal in homozygous mutant testes (**Supporting Figure S1B**). These results are similar to our initial survey of expression (**Figure 2**). Taken together, these results suggest that the targeted allele does not express the full complement of transcripts generated by the wild-type allele. In addition, no MUC16 protein was detected by immunofluorescence using a polyclonal antibody (**Figure 3**). With respect to the testis, the targeted modification is clearly a loss-of-function allele. Formal demonstration that the targeted mutation is a null allele in the testis is hampered because *Muc16* is a very large gene with many exons [22].
