# Supplementary material for: CA125/MUC16 Is Dispensable for Mouse Development and Reproduction
Source: PLoS One. 2009 Mar 5;4(3):e4675. doi: 10.1371/journal.pone.0004675 (PMC2650410; doi:10.1371/journal.pone.0004675)
Supplement: Table S1 — Summary of PCR primer sequences. (0.04 MB DOC) [file pone.0004675.s002.doc]

Supporting Table S1. Summary of PCR primer sequences

| Primers | Sequence of forward(F) and reverse(R) primers | Annealing  temperature  (°C) | Accession # |
| --- | --- | --- | --- |
| 1F  2F  3F  3F-2  3R  3R-2  del3R  4R  4F  5R  5R-2  5F  6R | 5’-CACTGGACTCTGGTACTGTGC-3’  5’-CAACGACAGGAGTCCTGGAAG-3’  5’-CAGCATTTCCTATCCAATCACTAACCAG-3’  5’-CCCAAGACAAACACAAGAAAGTTG-3’  5’-CCTGGTTAGTGATTGGATAGGAAATG-3’  5’-CTTCCTGGGCCTCATAGGGAC-3’  5’-CATGGGCACTTCAGTATGGTTGAGAC-3’  5’-CAGTGTCTCCATGGACAGCTCC-3’  5’-GAGCTGTCCATGGAGACACTG-3’  5’-CCTCTGTGGTTAGATCTTTATGGTTAGC-3’  5’-GCTGAAGTAATCTCTAAAGCATGG-3’  5’-GGATAAGACTACAACTTCACTACATG-3’  5’-CCAATACTGCTATTCTTGAACATGGGC-3’ | 54  56  56  54  55  56  58  57  55  58  55  53  58 | XM_911929 |
| lacZ_F  lacZ_R | 5’-GCATCGAGCTGGGTAATAAGGGTTGGCAAT-3’  5’-GACACCAGACCAACTGGTAATGGTAGCGAC-3’ | 64  62 | CP_000800 |
| neo_F | 5’-CAGGACATAGCGTTGGCTACC-3’ | 56 | EU_530629 |
