# Supplementary figures and images for: CA125/MUC16 Is Dispensable for Mouse Development and Reproduction
Source: PLoS One. 2009 Mar 5;4(3):e4675. doi: 10.1371/journal.pone.0004675 (PMC2650410; doi:10.1371/journal.pone.0004675)

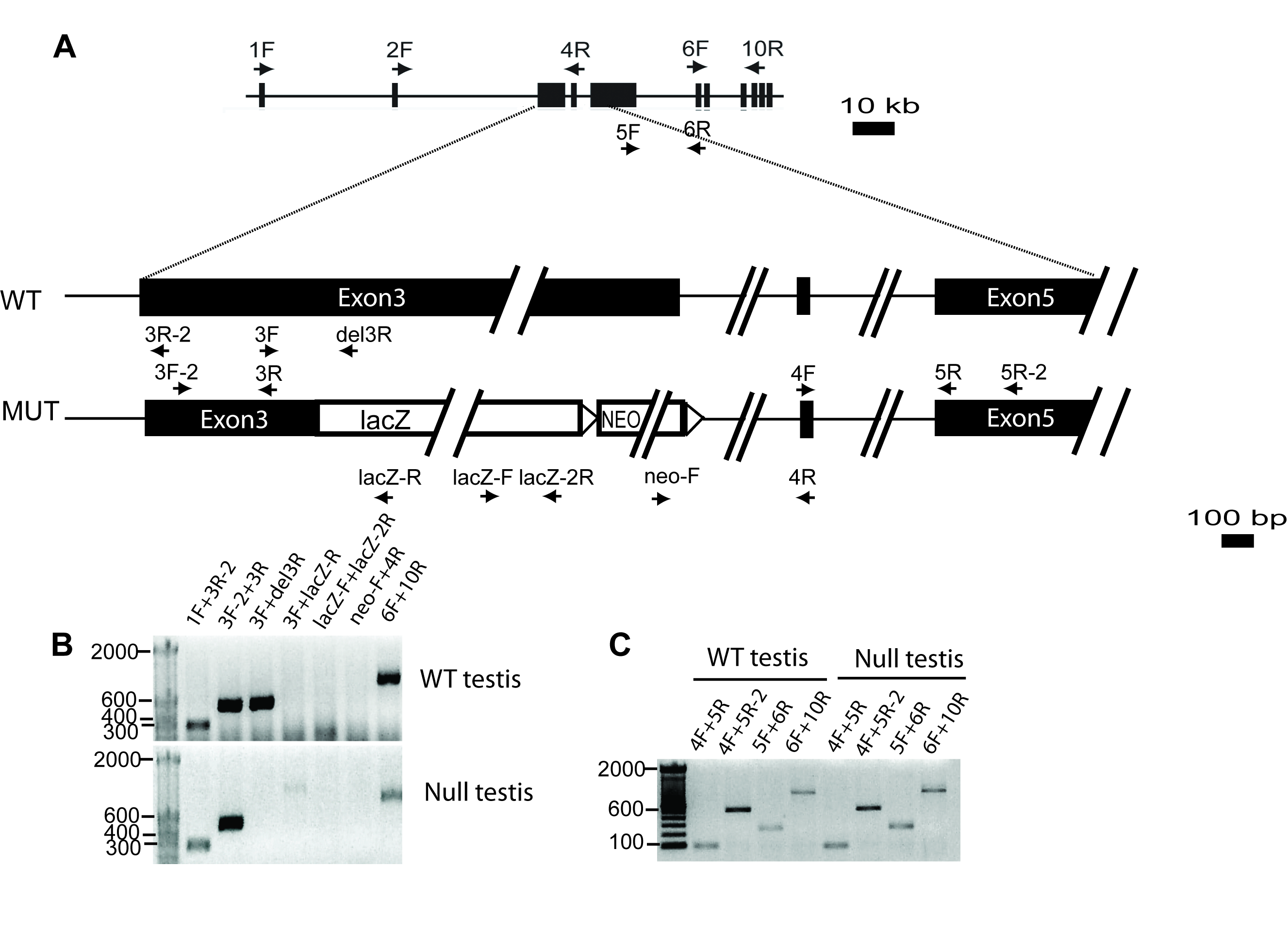

Supplement: Figure S1 — RT-PCR analysis of Muc16 locus. (A) Organization of the mouse Muc16 gene and location of primers used for RT-PCR analysis (indicated by arrows). (B, C) RT-PCR analysis of Muc16 expression upstream and downstream of the targeted region in adult Muc16 wild-type and null testis. Forward (F) and reverse (R) primers are indicated. (2.08 MB TIF) [file pone.0004675.s003.tif]
